# Cellular Pathogenesis of Chemotherapy-Induced Peripheral Neuropathy: Insights From *Drosophila* and Human-Engineered Skin Models

**DOI:** 10.3389/fpain.2022.912977

**Published:** 2022-07-08

**Authors:** Grace Ji-eun Shin, Hasan Erbil Abaci, Madison Christine Smith

**Affiliations:** ^1^Zuckerman Mind Brain and Behavior Institute, Jerome L. Greene Science Center, Columbia University, New York, NY, United States; ^2^Department of Dermatology, Columbia University Medical Center, Saint Nicholas Avenue, New York, NY, United States

**Keywords:** CIPN, *Drosophila*, human skin model, neuropathic pain, nociception and pain, tissue-engineered skin, chemotherapy

## Abstract

Chemotherapy-induced peripheral neuropathy (CIPN) is a highly prevalent and complex condition arising from chemotherapy cancer treatments. Currently, there are no treatment or prevention options in the clinic. CIPN accompanies pain-related sensory functions starting from the hands and feet. Studies focusing on neurons *in vitro* and *in vivo* models significantly advanced our understanding of CIPN pathological mechanisms. However, given the direct toxicity shown in both neurons and non-neuronal cells, effective *in vivo* or *in vitro* models that allow the investigation of neurons in their local environment are required. No single model can provide a complete solution for the required investigation, therefore, utilizing a multi-model approach would allow complementary advantages of different models and robustly validate findings before further translation. This review aims first to summarize approaches and insights from CIPN *in vivo* models utilizing small model organisms. We will focus on *Drosophila melanogaster* CIPN models that are genetically amenable and accessible to study neuronal interactions with the local environment *in vivo*. Second, we will discuss how these findings could be tested in physiologically relevant vertebrate models. We will focus on *in vitro* approaches using human cells and summarize the current understanding of engineering approaches that may allow the investigation of pathological changes in neurons and the skin environment.

## Introduction

The discovery and optimization of chemotherapy has significantly contributed to an increase in survival for cancer patients in recent decades (1, 2). Despite the increasing discovery of alternative treatment options in cancer treatment, chemotherapy remains a major first-line therapy for most cancer patients (3–6). With increasing survival rates, adverse sequelae of chemotherapeutic treatment are a significant burden to patients, caregivers, and society (7). Chemotherapy-induced peripheral neuropathy (CIPN) is one of the major side effects of chemotherapy, affecting up to over 60% of patients in short-term and long-term, more than 2 years after cessation of treatment (8–13). CIPN is predominantly sensory, but motor and autonomic symptoms are also reported. Symptoms include pain-related sensory dysfunction, starting in the hands or feet. CIPN severely reduces patients' quality of life in the long term and can adversely affect patients' survival by limiting required chemotherapy treatment (8, 14). Despite active investigation, the underlying pathological mechanisms are still unclear, and treatment or prevention strategies are unavailable in the clinic (8–10, 15, 16).

The most commonly used chemotherapeutic agents can be classified into six different groups: taxanes (paclitaxel, docetaxel), platinum-based antineoplastics (oxaliplatin, cisplatin, carboplatin), proteosome inhibitors (bortezomib, carfilzomib, ixazomib), vinca alkaloids (vincristine, vinblastine, vinorelbine), epothilones (ixabepilone), and immunomodulatory drugs (thalidomide, pomalidomide, lenalidomide) (17, 18). Mechanisms of how these chemotherapeutic agents induce apoptosis of cancer cells are reviewed elsewhere (17, 18). As these chemotherapeutic agents target diverse cellular pathways, agent-specific and common symptoms are reported. Cumulative doses are strongly correlated with CIPN severity (19). Common symptoms affecting somatosensation include paresthesia (abnormal sensation) such as tingling and pins and needles, numbness, and pain, which usually start from the hands and feet and may accompany mild motor neuropathy (19, 20). In addition, diverse pain-related symptoms are reported, including allodynia, hyper or hypoalgesia, burning or shooting pain, or pain that may be more painful than original cancer (20, 21). Of these patients, ~30% of patients develop pain (21, 22). Currently, there are no prevention or approved treatment options in the clinic, and the clinical intervention is limited to symptomatic relief provided by opioid analgesics, antidepressants, or anticonvulsants (11, 23, 24). Duloxetine is the only approved option for CIPN, and clinical trials have shown to reduce the pain severity in a subpopulation of affected patients (25, 26). Other options include gabapentin, with limited efficacy, while it is an effective option for other types of peripheral neuropathy (17). Sex-specific sensitivity of CIPN is debatable, however, females showed a higher risk for the development and severity in rodent models (27–29).

Extensive studies were performed to elucidate underlying pathological mechanisms, identify biomarkers, develop standard clinical assessments, and identify and validate cellular and genetic targets that may prevent or reverse CIPN (17, 30, 31). Many promising results from these preclinical studies exist, yet no treatments are currently approved for use in the clinic. An incomplete understanding of the cellular and molecular mechanisms may underlie these unmet needs (8–11). As sensory neurons are primary targets of many chemotherapeutics, many studies have focused on neuron intrinsic mechanisms underlying CIPN pathology. These studies reported transcriptional changes in DRGs, structural changes in axons, and their terminals within the epidermal layer. Notably, a strong correlation was shown between intraepidermal nerve fiber density (nociceptive neuron terminals residing in the epidermis) and the CIPN severity in both patients and animal models (32). However, nerve fiber density does not correlate with painful symptoms (33–36). These studies have identified several common pathological features from various types of chemotherapeutics. Common features include length-dependent axon degeneration, mitochondrial dysfunction, oxidative stress, epidermal cell and the extracellular matrix (ECM) degeneration, and immune activation (10, 15, 37–41). Neuron-intrinsic pathways mediating survival (41–47) and transcriptional and translational changes (48–52) have also been demonstrated as critical mechanisms of CIPN pathology.

Chemotherapeutics can also directly affect neighboring non-neuronal cells. Recent studies highlight the importance of neuronal interactions with their environment in both peripheral and central environments involving the immune system, Schwann cells, and epidermal layers of the skin (10, 53–65). Insights from these studies point to the importance of studying neuronal toxicity in the context of the local environment, yet how neurons interact with non-neuronal cells is only beginning to be understood and mostly unknown in the context of CIPN. To understand intercellular mechanisms, *in vivo* models or *in vitro* models that allow manipulation and monitoring of multiple cell types are required. Several recent CIPN studies have demonstrated *Drosophila melanogaster* as a simple and genetically tractable *in vivo* model that could fill knowledge gaps with conserved CIPN phenotypes and examples with successful translation to other mammalian models (42, 43, 66–72). Utilizing full advantages of *Drosophila* models as a part of multi-model approaches with physiologically relevant vertebrate models, such as rodent models or human engineered skin models, may provide significant insights into a deeper understanding of CIPN pathology.

## Experimental Approaches to Study CIPN Mechanisms

Previous research on CIPN has primarily been conducted *in vitro* and *in vivo* using rodents (73), with the initial study of CIPN in rats in 1992 using cisplatin, a platinum-based chemotherapeutic (74). A recent systematic analysis of the literature on CIPN models discovered that, of 183 different models, 12 species were used, and 85.2% of these studies were conducted in rodents (73). The study then ranked the models in their efficacy in representing CIPN, as evaluated by assessing mechanical allodynia, thermal hyper and hypoalgesia, histological damage to the peripheral nervous system, and functional neurophysiological changes (73). The most efficacious models were determined to be rats and mice; however, the study also recognized that more research is needed in other potentially effective models, including *Drosophila* and zebrafish, which showed a consistent efficacy in driving CIPN phenotypes using paclitaxel, cisplatin, and bortezomib (65, 73, 75).

Rodent *in vivo* models of CIPN are typically adult mouse and rat models injected with chemotherapeutics via intraperitoneal or intravenous injections, mimicking chemotherapeutic treatment for patients to recapitulate pharmacodynamics and pharmacokinetics (73, 76). Typical readouts of these models include genomic changes in DRGs, axon and axon terminal (intraepidermal nerve fiber), electrophysiological, and behavioral changes. While CIPN affects all sensory modalities, these animal models focus on investigating the changes in nociception and measure pain phenotypes in response to noxious stimuli of different modalities: mechanical, thermal, and cold stimuli (76). Although rodent studies significantly facilitated our understanding of CIPN pathology, more studies demonstrated molecular differences between human and rodent DRGs (77), which is predicted as a roadblock to effective translation. Accordingly, efforts are made to understand how insights gained from rodent models can be effectively translated into humans. More recently, human models utilizing induced pluripotent stem cell (iPSC) approaches (78–80) were also used in the field, and limited non-human primate models (73) have been used to validate findings from small animal models.

Non-mammalian *in vivo* models, *Drosophila*, zebrafish, and *C. elegans* provide advantages in genetics and live monitoring of neuron phenotypes and the surrounding environment (75). While sensory neurons in non-mammalian models have several differences from mammalian counterparts, these models are advantageous and complementary to mammalian models. Several studies from *Drosophila* and zebrafish have been validated in rodent models [reviewed in (75)], however, their efficacies have not yet been tested in humans. Considering the knowledge gaps in CIPN pathology on how cellular environment (both cancer and cancer-free) influence neuronal health, these simple models may serve as effective tools for advancing our understanding of CIPN pathology and identifying candidates for CIPN treatment.

For *in vitro* studies, rodent primary sensory neurons (embryonic and adult), immortalized sensory neurons, non-neuronal cells, and cells and tissues from other non-conventional models, including Aplysia and squid, were used (15, 81, 82). To address the limitation of *in vitro* approaches, several studies deployed co-culture models to investigate inter-cellular mechanisms in CIPN (83–86). Additionally, studies that used compartment culture extended our understanding of an axon-specific vulnerability of the sensory neurons. Several *in vitro* studies using compartmentalized cultures demonstrated that axon terminals, rather than cell bodies, are sensitive to paclitaxel toxicity (45, 87, 88). These studies consistently showed a reduction in axon length only after the paclitaxel addition in the axon compartment, highlighting that paclitaxel induces axon degeneration through local mechanisms in the sensory neuron's peripheral environment. A combination of co-culture and compartment culture approaches may be useful in future studies to simulate cellular interactions of axon terminals and non-neuronal cells.

## *Drosophila* Models of CIPN

There are only handful of *Drosophila* CIPN studies so far, however, the use of chemotherapeutics in *Drosophila* models for studying cancer has been well established in the field (89–91), with over 400 articles in PubMed at the time of writing this review. A PubMed search on “*Drosophila* AND CIPN” resulted in 9 articles, including seven primary articles. The second search using “*Drosophila* AND neuron AND (paclitaxel OR vincristine OR bortezomib OR cisplatin)” resulted in 9 additional articles. Three of these studies from the second search were relevant (70, 71, 92) and included in this review. An additional manual search revealed another article in cisplatin-induced peripheral neuropathy (93). Overall, *Drosophila* CIPN studies focused on three common chemotherapeutics, paclitaxel (42, 43, 72, 94), cisplatin (66, 67, 70, 71, 92), and bortezomib (69). Three of these studies (42, 69, 72) used *Drosophila* as a part of multi-model studies. Other studies have discussed conserved mechanisms shown in other mammalian CIPN studies in the literature (43, 66–68, 70, 71) (Table 1).

**Table 1:** Summary of *Drosophila* CIPN studies.

**Study**	**Major findings in *Drosophila***	**Translation to vertebrate models Evidence from the literature**
**Paclitaxel**		
Bhattacharya et al. (42)	° Chronic treatment of paclitaxel induced nociceptive neuron degeneration ° Retinophilin knockdown prevented paclitaxel-induced degeneration in nociceptive neuron dendrites and axons and severed olfactory axons. ° Overexpression of Nmnat, but not p35, prevented paclitaxel-induced degeneration in nociceptive neurons	°Subsequent experiments in embryonic mouse DRG culture demonstrated a conserved role of MORN4 in axonal degeneration following axotomy ° Retinophilin (MORN4) and Nmnat are conserved in mammals ° Nmnat in mammalian CIPN models showed protective effect
Brazill et al. (43)	°Acute treatment of paclitaxel induced dose-dependent hypersensitivity, hyperbranching, and perturbation to microtubule organization ° Overexpression of Nmnat prevented paclitaxel-induced hypersensitivity, but not hyperbranching or microtubule organization phenotypes	°Nmnat in mammalian CIPN models showed protective effects
Kim et al. (68)	°PINK1 overexpression changed nociceptive neuron dendrite morphology and levels of PINK1 determined sensitivity to noxious stimuli ° PINK1 overexpression protected oxidative stress in mitochondria induced by paclitaxel	°PINK1 is a conserved gene in mammals and showed protective effects in Parkinson's disease
Shin et al. (72)	°Chronic treatment of paclitaxel induced dose-dependent nociceptive neuron degeneration, altered branching pattern, and hyposensitivity ° Paclitaxel perturbed trafficking of integrins, recycling endosomes and lysosomes ° Acute treatment of paclitaxel induced trafficking phenotypes prior to degeneration in nociceptive neurons ° Overexpression of integrins in nociceptors protected against selected paclitaxel-induced phenotypes	° Paclitaxel reduced membrane recycling of integrins in mouse DRG neurons ° Paclitaxel perturbed motility of recycling endosomes and lysosome prior to degeneration in mouse DRG neurons ° Transduction of human ITGB1 in adult DRG neurons prevented degeneration in adult mouse DRG neurons ° Levels of integrins correlate with capacity of neuron regeneration after injury
**Cisplatin**		
Podratz et al. (93)	°Acute cisplatin treatment induced dose-dependent lethality, reduced geotactic climbing behavior, cisplatin-DNA binding, and cellular apoptosis in brain, ovaries, but not in kidney and heart ° p35 overexpression prevented cisplatin-induced apoptosis in the brain and restored climbing behavior	°Platinum-adduct levels found to be comparable to rat DRG neurons in their previous study
Podratz et al. (70)	°Acute treatment of cisplatin reduced mitochondrial activity, increased reactive oxygen species production and mitochondrial pausing° Cisplatin treatment resulted in behavioral deficiencies (heat sensing and righting) ° Overexpression of p35 prevented behavior and phenotypes	° Mitochondria phenotypes are consistent with and complement the findings in mouse DRG neurons in their previous study
Groen et al. (66)	°Common background strains (Oregon-R, Canton-S, w^1118^) have different sensitivities to cisplatin in climbing behavior and survival rate ° ABC transporter mutants (relating to eye color mutants) have increased sensitivity to cisplatin	°ABC transporters have been linked with cisplatin efficacy and multi-drug resistance
Groen et al. (67)	°Flies harboring attp40 insertion site have reduced ND-13A expression, a part of the mitochondria electron transport chain complex I° Neuron-specific ND-13A knockdown specifically prevents neuronal apoptosis (but not ovary cells), climbing deficiencies, and oxidative stress ° Protective capacity of ND-13A is Sirt-1 and PGC1-alpha dependent, and overexpression of Sirt-1 strongly prevented cisplatin phenotypes	°SIRT1 activation protected sensory neurons from cisplatin-induced peripheral neuropathy in rodent models
**Bortezomib**		
Pero et al. (69)	°Chronic treatment of bortezomib induced degeneration in nociceptive neurons° Acute treatment of bortezomib reduced catastrophe, rescue/nucleation frequencies and comet density by 3 h and reduced growth rate by 6 h	° Bortezomib induced degeneration and acutely perturbed microtubule dynamics in cultured adult mouse DRG neurons ° Bortezomib promoted accumulation of hyperstable forms of tubulin (Delta 2) in rodent and human tissues

### Sensory Neurons and Their Extracellular Environment in *Drosophila*

The peripheral nervous system (PNS) of larval *Drosophila* consists of somatosensory neurons of different modalities, stereotypically localized along the basal surface of the epidermis (95). The PNS of *Drosophila* is organized segmentally and sensory and motor neurons are organized in a stereotyped pattern. Sensory neurons are categorized into two groups, type I neurons with ciliated monopolar dendrites and type II neurons with multiple dendrites. Multiple dendritic (md) neurons are further categorized into three subtypes: tracheal dendrite (md-td), bipolar dendrite (md-bd), and dendritic arborization (md-da) neurons (96, 97). Md-da neurons display class-specific morphologies in their innervation along the body wall (97). Classes I-IV md-da neurons have different sensory modalities, proprioception (cI), touch (II, III), cold nociception (III), mechanical and thermal nociception (IV) (98–101). Of particular interest to CIPN studies are class IV nociceptive neurons, highly branched somatosensory neurons that innervate the epidermis, running between the muscles of the body wall and the epithelium using intricate space-filling arbors (97, 98). Major differences between *Drosophila* and mammalian sensory neurons include location of cell bodies (43) and microtubule orientations in dendrites, which are sensory neuron terminals in the periphery, analogous to vertebrate sensory afferents in the dermal and epidermal layers (102).

Despite these differences, *Drosophila* nociceptive neurons share key features with their mammalian counterparts, such as naked nerve endings contacting the epidermis and ongoing terminal and substrate remodeling in mature stages (98, 103–107). Similarly, the Drosophila immune system is comprised of an evolutionarily conserved innate immune system with specialized immune cells analogous to vertebrate macrophages (108–111). They likewise surround nociceptive neurons and become robustly activated following wasp attack and injury (108–110, 112, 113), homologous to skin-residing macrophages, which actively patrol local nerves and are required for nerve regeneration after injury (114). Thus, *Drosophila* may serve as an effective system to unravel the complex cellular and molecular basis of interactions between nociceptive neurons and extrinsic factors and the relevance to sensory pathology.

### *Drosophila* as a Model to Study Peripheral Neuropathy

Human genes related to pain, TRPV, TRPA1, TRPM2, PIEZO1, PIEZO2, and ASIC3, are conserved in *Drosophila* (115, 116). Many *Drosophila* studies have significantly contributed to our understanding of pain and neurodegeneration. For example, *Drosophila* models identified the first transient receptor potential (Trp) channel (117) and enabled discovery of conserved axon death pathway involving Toll receptor adaptor Sarm (sterile α/Armadillo/Toll-Interleukin receptor homology domain protein) (118), a key candidate for CIPN prevention (44–47).

The advantages of *Drosophila melanogaster* models include genetic amenability, a shorter life cycle, and a large number of offspring (119). Widely available genetic approaches that specifically mark and manipulate nociceptive neurons are powerful tools for understanding cellular and intracellular changes (120). For example, the promoter region isolated from pickpocket (ppk) gene, which encodes a degenerin/epithelial sodium channel subunit, is often used for specific labeling and manipulation of class IV neurons (121, 122). Using binary systems such as Gal4-UAS and LexA-LexAOp systems (123), potential genes of interest can be either knocked down or overexpressed using readily available transgenic lines. Because 60–75 % of human disease-related genes are predicted to have orthologs in *Drosophila* (124, 125), abundant *Drosophila* toolkits provide effective approaches to study genes that are responsible for nociception and peripheral neuropathy.

The functional role of class IV neurons was first demonstrated by identification of TrpA1 homologue *painless* (126) and subsequent demonstration of nocifensive behavior in response to noxious thermal and mechanical stimuli (98). Damage to these nociceptors is linked to sensory dysfunction based on nociceptive behavioral studies in *Drosophila* models and corresponding changes in unmyelinated nociceptive sensory endings in response to chemotherapy treatment (42, 43, 68, 72). Behavioral nociceptive assays conducted in the field typically evaluate nocifensive behavior, analogous to stimulus-evoked responses in rodent models using Von Frey test (mechanical nociception) or the Hargreaves test (thermal nociception). In response to noxious thermal or mechanical stimuli, *Drosophila* larvae show a stereotyped behavioral response including C shape bending followed by lateral rolling behavior and fast crawling (98, 126). Other behavioral studies have assessed sensory-motor circuit function by evaluating geotactic climbing behavior and righting behavior (the ability to flip back onto the ventral side following placement on the dorsal side) (70, 71, 93).

### CIPN Study Design and Readouts in *Drosophila* Models

*Drosophila* CIPN models found similar degenerative phenotypes to mammalian models in response to treatments with the commonly prescribed drug paclitaxel and bortezomib (42, 69, 72). In addition, *Drosophila* models of CIPN also demonstrate comparable pathological progression in terms of the dose and duration dependence of hyposensitivity (72) or hypersensitivity (43, 70) in response to noxious thermal stimuli (43, 70, 72).

Studies investigating CIPN in *Drosophila* have so far mainly focused on understanding chemotherapy-induced changes of neurons *in vivo*. This includes investigating morphological changes, chemotherapeutic-induced intracellular phenotypes in mitochondria and endosomes, and, in some studies, correlating these with behavioral changes (42, 43, 68–72). Leveraging a deep understanding of nociceptive neuron morphology in *Drosophila*, studies have characterized specific changes in branch pattern, dynamics, and degeneration (43, 72), adding to our understanding of mechanisms of neuronal changes in CIPN pathology. Both chronic (42, 69, 72) and acute treatments (43, 66, 68–72) have been conducted to assess short and long-term toxicity of chemotherapeutics. As shown in other mammalian studies, *Drosophila* CIPN models also showed dose-dependent phenotypes in nociceptive behavior and sensory neuron morphology (43, 66, 70–72).

Due to transparent body that allows live imaging and extensive understanding of nociceptive neuron development, maintenance, and a stereotyped nocifensive behavior, *Drosophila* larval sensory neurons have been a preferred CIPN model over adult flies in the field. Therefore, potential target genes and pathways identified in the model should be validated in adult models, such as adult mouse *in vitro* and *in vivo* models. In *Drosophila* models, chemotherapeutic agents had been fed on an *ad libitum* basis, an experimental design that conforms with constant feeding behavior (127) at larval stage and was also proved to be effective in adult models. While physiological concentrations of chemotherapeutics are unknown, studies have consistently reported dose-dependent phenotypes suggesting that the drug delivery is effective. Future studies quantifying tissue concentration of chemotherapeutics in these feeding paradigms will further facilitate translation of *Drosophila* studies.

### Potential CIPN Mechanisms Identified in *Drosophila* Models

#### Paclitaxel-Induced Peripheral Neuropathy Models

Paclitaxel is one of the most commonly used chemotherapeutic agents, frequently used to treat solid tumors, including breast, ovarian, lung, gastric, and head and neck cancers (15, 128, 129). Paclitaxel binds to β-tubulin in the tubulin polymer along the lumen of microtubules, suppressing dynamics and promoting tubulin polymerization (130–132). Up to 80% of patients treated with paclitaxel develop peripheral neuropathy and a subset of these patients have neuropathic symptoms in the long term (133). Mechanisms of CIPN arising from paclitaxel have been extensively studied, however, unifying pathological mechanisms are debatable, and definitive answers to PIPN mechanisms that can be translated into reliable methods for clinical interventions are unavailable.

The paclitaxel-induced neurotoxicity model in *Drosophila* started from the work of Bhattacharya and colleagues (42). The study used paclitaxel treatment as a method for neuronal injury and found that paclitaxel treatment results in axonal swelling and fragmentation without apoptosis. This study hypothesized that loss-of-function of a gene in the axonal degeneration pathway would delay or prevent degeneration of neurons following an injury and conducted RNAi screen. The screen consisted of 490 genes with enzymatic functions and known to function in the nervous system using pan-neuronal driver. Consequently, the screen identified MORN (membrane occupation and recognition nexus), encoded by retinophilin, a gene previously reported to function in the retina and store-operated calcium release leading to phagocytosis in macrophages in *Drosophila* (134–136). Additionally, they discovered that paclitaxel-induced degeneration of dendrites and axons could be prevented by overexpression of Nmnat (nicotinamide mononucleotide adenylyltransferase) or the knockdown of MAP3K Wallenda, orthologous to DLK in mammals. The study further showed that the knockdown of mouse ortholog of retinophilin, MORN4, prevented axonal degeneration following axotomy of embryonic mouse DRG neurons, suggesting conservation of protective capacity in mammalian system. This study highlights the utility of *Drosophila* model as an *in vivo* screening system in identifying conserved genes that may harbor therapeutic potential for CIPN treatment.

Severe degeneration of distal and proximal axons shown in the above study (42) likely represent late pathological stages of CIPN. Another study by Brazill and colleagues complemented the initial paclitaxel study by modifying paclitaxel feeding regimen including lower dosages (10–30 μM) and shorter treatment time (up to 48 h) (43). This approach enabled characterizing early pathological changes by paclitaxel. The authors observed an increased density in nerve endings, instead of severe degeneration following paclitaxel treatment. The branch phenotypes were correlated with decreased dynamics of nerve endings, particularly resulting in inhibition of dendrite terminal retraction. The study further identified disrupted neuron-specific microtubule associated proteins, MAP1B/Futsch, providing a potential molecular mechanism underlying changed dynamics of dendrite terminals. These morphological changes were accompanied by hypersensitivity to thermal noxious stimuli in a dose-dependent manner. This study further demonstrated that overexpression of the neuronal maintenance factor, Nmnat, mitigates hypersensitivity without affecting hyperbraching or Futsch disruption (43). This may indicate uncoupling of “form and function” whereby function is selectively mediated by NMNAT (43). Alternatively, it could indicate a partial protection of neuropathic phenotype by Nmnat overexpression and may require further behavioral testing of additional types of sensory dysfunction such as allodynia to delineate underlying mechanism.

Another study investigated the therapeutic potential of a conserved gene involved in mitochondria quality control in a *Drosophila* CIPN model (68). A mitochondrial Serine/Threonine kinase, PINK1 (Phosphatase and tensin homologue-induced putative kinase 1), has been shown as a molecular sensor for mitochondrial damage and shown to be involved in key steps in mitochondria quality control (137, 138). PINK1 mutations cause early-onset autosomal recessive Parkinson's disease, whereas its expression protects against various toxic insults in models of Parkinson's disease (137). Kim and colleagues (68) found that ectopic PINK1 expression prevented an increase in mitophagy-related oxidative stress in *Drosophila* class IV neurons upon paclitaxel treatment. In parallel, PINK1 levels determined the morphology and thermal sensitivity of cIV neurons, suggesting that PINK1 may be a critical component of cIV neuron development and maintenance. As knockdown of PINK1 specifically reduced branching and thermal sensitivity but not the baseline levels of mitophagy, this finding suggests that PINK1 has dual roles in nociceptive neuron health and maintenance.

A recent study reported cellular mechanisms of paclitaxel toxicity mediated by endocytic recycling pathway and identified cell surface receptors integrins as a conserved gene that prevents CIPN pathology (72). This study employed a complementary approach combining two established CIPN models, *Drosophila* sensory neurons and primary DRG mouse neuron cultures. The authors found that chronic treatment of *Drosophila* larvae with paclitaxel (10–20 μM) resulted in degeneration and morphological alteration of the branching patterns of nociceptive neurons. As the altered branching patterns resembled phenotypes when neuron-substrate interactions are perturbed, this study further investigated the potential protective capacity of integrins, key cell surface receptors known to maintain interactions between neurons and the ECM (106, 107). Upon overexpression of integrins in nociceptive neurons, both degeneration and branching pattern phenotypes were significantly reduced. These morphological changes corresponded to reduced nociceptive responses to noxious thermal stimuli, which was also prevented by cell-specific overexpression of integrins. Given the critical role of integrins in development and maintenance mediated through interactions with the extracellular environment (106, 107), this strongly points to the importance of neuron-substrate relationships in neuronal health and function in CIPN. Furthermore, this study proposed endosomal changes underlying paclitaxel-induced changes in nociceptive neurons. Paclitaxel treatment reduced endosome-mediated trafficking of integrins. Super-resolution and live imaging of animals with acute and chronic paclitaxel treatment revealed that impaired recycling pathways involved in integrin membrane trafficking preceded morphological degeneration. Using mouse DRG neurons, the study further validated that endocytic changes precede axon degeneration and that integrin overexpression of human integrin beta-subunit 1 (a major beta subunit of integrin heterodimers in mammals) effectively prevented degeneration following paclitaxel treatment. Because surface expression of integrins is required in neuronal interactions with epidermis and the ECM, these results highlight the importance of neuron-substrate interaction in CIPN pathology that is controlled by endocytic recycling pathways of cell surface proteins.

#### Cisplatin-Induced Peripheral Neuropathy Models

Cisplatin is a platinum-based drug that binds to mitochondrial and nuclear DNA and creates intra-strand cross-links forming platinum-DNA adducts. Accumulation of these adducts causes DNA damage and leads to apoptosis. Similar to paclitaxel, it is used to treat solid cancers such as lung, breast, ovarian, and colon cancers; however, unlike paclitaxel, its effect is not cell cycle-specific. Cisplatin is notably toxic to neurons (17). Cisplatin can adversely affect sensory nervous system, by causing apoptosis of somatosensory neurons and hair cells, leading to permanent sensory loss (17) and ototoxicity (92, 139).

A series of studies from the Windebank lab have pioneered *Drosophila* larval and adult models of cisplatin-induced peripheral neuropathy (66, 67, 70, 71, 93). The initial study in the lab used adult fly model to study the effect of cisplatin in neurons in the central nervous system and geotactic climbing behavior (93). The authors found that acute feeding of cisplatin (10–200 μg/mL) dose-dependently caused lethality and climbing defects, which was prevented by overexpression of p35, a pan-caspase inhibitor and anti-apoptotic protein. The group further demonstrated that cisplatin also causes similar toxicity in the larval model (70). Larval *Drosophila* fed with 10 and 25 μg/mL cisplatin showed a deficit in both motor and sensory behaviors: hypersensitivity to heat and attenuated motor-proprioceptive behavior. P35 overexpression also ameliorated these larval behavior phenotypes, suggesting that p35 may be an attractive target for cisplatin-induced peripheral neuropathy. This investigation further identified cisplatin-induced mitochondrial phenotypes in *Drosophila* motor neurons, following from their earlier study in a rodent CIPN model that demonstrated cisplatin-induced mitochondrial damage in DRG neurons (140). Using *Drosophila* as an *in vivo* model to study mitochondrial function and axon transport, the study demonstrated specific mitochondrial defects by cisplatin treatment: cisplatin reduced mitochondrial activity and mitochondrial membrane potential, whereas it increased reactive oxygen species production and mitochondrial pausing (70). As cisplatin-induced peripheral neuropathy is predominantly sensory, future studies investigating sensory neuron phenotypes in *Drosophila* would provide additional insights into pathological mechanisms of cisplatin-induced toxicity.

Susceptibility of different strains to platinum-based drugs have been reported in rodent models, which may explain patient-specific susceptibility to these drugs (141, 142). To provide underlying mechanisms, two additional studies utilized different *Drosophila* strains to examine and investigate potential genetic markers for CIPN susceptibility (66, 67). These studies identified ABC transporter (linked with a common control *Drosophila* strain w^1118^) and ND-13A, a component of the mitochondria electron transport chain complex I (linked with a transposable element insertion site attp40 in some *Drosophila* transgenic lines). Consistent with the results from rodent studies, these studies provide critical information for effective experimental designs for CIPN studies and whether these strains confer different susceptibilities to other chemotherapeutic drugs should be investigated in future. Furthermore, these studies highlight the utility of *Drosophila* CIPN models to uncover novel genes responsible for patient susceptibility and treatment target pathways in CIPN.

#### Bortezomib-Induced Peripheral Neuropathy Model

Bortezomib is a type of proteosome inhibitor used routinely to treat multiple myeloma, mantle-cell lymphoma, and amyloidosis. Inhibition of proteosome activity leads to the misfolded protein accumulation and apoptosis of cancer cells (143). Up to 80% of newly-diagnosed patients with bortezomib treatment develop peripheral neuropathy, however, in the majority of cases, Bortezomib-induced peripheral neuropathy can be resolved by drug cessation or dose reduction (144).

Bortezomib-induced peripheral neuropathy mechanisms have not yet been extensively studied in *Drosophila*; however, the *Drosophila* model was employed in a multi-model study consisting of mouse, rat, zebrafish, *Drosophila*, and human (69). Consistent with other models in the study, *Drosophila* larvae fed with bortezomib showed degeneration of nociceptive neuron terminals after chronic treatment and perturbation of microtubule dynamics after acute treatment. As an *in vivo* model amenable for live imaging in an intact animal, the *Drosophila* model provided a complementary result to *in vitro* rodent models and human tissues, corroborating the underlying mechanisms of bortezomib toxicity via microtubule stabilization (69).

#### Limitations and Future Potentials of *Drosophila* CIPN Models

Although not yet widely used, studies using *Drosophila* CIPN models proved to be relevant and useful in discovering potential treatment targets and underlying pathological mechanisms. Like rodent models, *Drosophila* CIPN models have so far primarily focused on neuron intrinsic mechanisms. *Drosophila* models provide established platforms to investigate neuronal interactions with their extracellular environment, particularly with epidermal cells and the ECM. Given the importance of neuronal interactions with epidermal keratinocytes in nociception, the *Drosophila* model will provide a simple *in vivo* model to inform how these inter-cellular interactions contribute to pathological progression of CIPN. Another area of interest may be combining the wealth of cancer studies in *Drosophila* into CIPN investigation. While cancer-bearing models would provide highly relevant microenvironment for studying CIPN mechanisms and validate safety and efficacy of CIPN treatment targets, it is challenging to generate vertebrate models that combine cancer and pain models. As an invertebrate system, *Drosophila* cancer models may provide an opportunity to investigate CIPN mechanisms in a relevant cancer environment. As shown in several studies in the field, future *Drosophila* CIPN studies should be designed in consideration with future or parallel rodent and human studies, preferably as a part of multi-model study to effectively demonstrate conserved mechanisms for CIPN pathology and contribute to prevention and treatment of CIPN.

## Possibilities of Utilizing Human-Engineered Skin Models in Understanding CIPN

Although animal models have provided many valuable insights into understanding CIPN pathology, CIPN models that capture human genetics and physiology would add significant advantages for clinical translation. There is no effective human model for studying CIPN to investigate neuropathic mechanisms in the context of their local environment. Utilizing human-engineered skin models may fill the knowledge gap to validate and further examine CIPN pathological mechanisms.

### Introduction to Human-Engineered Skin Models

The field of skin bioengineering has advanced significantly over the past several decades, offering physiologically-relevant models of human skin in different cellular and structural complexities (145). These advanced tissue-engineered skin (TES) models represent the 3D skin microenvironment and cellular diversity of human skin more closely compared to 2D cell cultures while still offering a large variety of molecular and cellular readouts. TES models are composed of different skin cell types self-assembled or reconstructed within a 3D hydrogel, typically collagen type I. Given that sensory terminals innervate the skin, CIPN research may significantly benefit from bioengineered human skin that emulates its native environment. The bioengineered 3D innervated skin models are expected to provide insights about the human relevance of findings obtained using animal models or simplified 2D models. Furthermore, through the capability of adjusting the complexity of bioengineered skin, this approach will provide an efficient model system to dissect the interactions of sensory neurons with different skin cell types.

There is a variety of commercially available full-thickness 3D skin models, which are typically composed of dermal fibroblasts and terminally differentiated layers of keratinocytes. The human skin is much more complex with more than 50 different cell types and several appendages, such as hair follicles, sebaceous glands and sweat glands. While our knowledge of the interactions between nociceptor sensory endings and the diverse cellular makeup in skin is limited, we are starting to understand interactions between the keratinocytes and nociceptive neurons (146, 147). For example, recent work suggested a determining role of specific epidermal cell populations on sensory neuron patterning and axonal growth. A recent study showed that a KRT17-positive subpopulation of keratinocytes residing in the follicular and interfollicular epidermis is required and sufficient for touch-sensitive sensory neuron patterning in mouse touch-domes (148). Arborization of neuronal axons is also mediated by various signal inputs in the extracellular space, including positive and negative ECM cues in the skin. Specialized ECM proteins play pivotal roles in axonal branching and patterning (149). For instance, EGFL6, a specialized ECM protein deposited by the epidermal stem cells in hair follicles influences the terminal anatomy of mechanosensory endings in the hair follicles (150). A growing number of studies show the interactions of sensory neurons with various skin cell types, such as endothelial cells, immune cells, and Schwann cells, during skin regeneration and inflammation. However, how skin cells play a role in the pathological progression of CIPN is mostly unknown. Therefore, the recapitulation of the cellular diversity in TES models is important to develop a physiologically-relevant CIPN model and understand pain-related sensory function.

Since Bell et al. introduced the first full-thickness TES in 1981 (151) with dermal fibroblasts and keratinocytes, there has been a substantial effort in incorporating other dermal and epidermal cell types. These include blood (152) and lymphatic endothelial cells (153) forming vascular networks, melanocytes producing pigmentation (154), and adipocytes generating the adipose-containing hypodermis compartment (155). In addition, there has been a significant interest in adding different immune cell types, such as macrophages, Langerhan cells, disease-specific effector T cells, dendritic cells, and neutrophils, into TES to generate immune-competent skin models (156). Moreover, skin appendages, i.e., hair follicles (157) and sweat glands (158), have recently been successfully integrated into TES, further increasing the functional and structural relevance of these models.

The current TES models are based on a reverse-engineering approach. Each primary or iPSC-derived cell type is expanded *in vitro* separately and then reconstructed in 3D for spontaneous self-organization of cells or assisted organization using engineered patterns to recapitulate cell-cell interactions. Therefore, it becomes technically challenging to include more and more skin cell types and components to eventually achieve an *in vivo* level complexity. However, in their current form, where several types of skin cells are included (fibroblasts, keratinocytes, and endothelial cells) together with nociceptors, they can still be invaluable models to dissect the interactions of nociceptors with multiple cell types in the context of CIPN and skin microenvironment. Given the spatial relationship between nociceptive neurons and the epidermis, such models are expected to serve as an effective platform for studying the neuron-ECM-epidermis interactions in a 3D environment with conserved physiological relevance to human skin.

Recent iPSC-derived skin organoids may address some of the challenges in TES regarding cellular diversity (159). The skin organoids approach mimics skin morphogenesis and simultaneously generates many skin cell lineages, including melanocytes, adipocytes, and hair follicles. In addition, it can generate specialized cell types, such as Merkel cells of the touch-dome, which cannot be incorporated into reconstructed TES models due to the difficulties of expanding these cells *in vitro*. Moreover, the presence of neurons and sensory neurons was demonstrated in skin organoids which may enable studying skin mechanosensation and pain. However, it is not yet known which subpopulations of sensory neurons exist in these organoids or whether they are mature enough to represent skin innervation and function. Nevertheless, despite its current limitations, the skin organoid is an exciting new approach that has the potential to be integrated into CIPN research in the future.

### Innervated TES Models

The proof-of-concept for incorporating sensory neurons into 3D skin has been demonstrated by several studies. Earlier studies integrated rat neurons isolated from dorsal root ganglia (DRG) into explanted human skin in a co-culture system (160). Later studies successfully innervated tissue-engineered skin with sensory neurons isolated from the mouse, rat, or porcine DRG or human iPSC-derived sensory neurons (161).

Roggenkamp et al. developed several 3D co-culture models with animal DRG sensory neurons and human fibroblast and keratinocytes from both healthy and diseased donors. In an early study (162), they seeded porcine DRG neurons embedded in collagen type I gel on a polyester/propylene matrix scaffold, and then added human TES composed of healthy dermal FBs and KCs. After 12 days of culture, neurites were observable in the dermal component with thin nerve endings ascending toward the epidermis resembling innervation *in vivo*. Using similar co-culture setup with skin fibroblasts and keratinocytes isolated from individuals with type II diabetes, the same group showed diabetic TES reduces porcine neurite outgrowth due to decreased levels of neurotrophic factors, illustrating the hypo-innervation in type II diabetes (163). In another disease model, they further showed that skin cells from atopic dermatitis patients promote neurite outgrowth in TES compared to healthy skin cells (164). These models demonstrated the capability to induce neurite outgrowth of sensory neurons in TES as a readout to assess disease-specific innervation mechanisms.

Another group induced mouse DRG ingrowth in TES and further tested the sensing functionality of the innervated model through topical application of capsaicin. The DRG neurons responded to capsaicin treatment by changes in Ca^2+^ influx. This study is significant in terms of showing neuronal functionality in TES (165).

A series of important papers from François Berthod's group broadened our understanding of the mechanism underlying the innervation of TES. In an early 2003 study (166), they used a collagen sponge populated with dermal fibroblasts and endothelial cells and highlighted that NGF is critical for neurite growth but not for the survival of mouse DRG neurons. In a separate study, they included mouse Schwann cells isolated into TES and showed that Schwann cells enhance the innervation process and can myelinate the DRG neurons in TES (167). This study is particularly important to show the proof-of-concept for sensory neuron myelination, a determining factor in achieving sensory function in TES. In two subsequent studies, the group implemented their approach, with slight modifications in the TES model, for aging (168) and wound healing applications (169). Their studies highlighted the importance of nerve-skin interactions demonstrating more efficient wound closure in the presence of sensory neurons through secretion of neuropeptide substance P.

The sourcing of human-derived DRGs has been a problem and recently been partially addressed by leveraging iPSC technology. By differentiating iPSCs into sensory neurons and Schwann cells, a fully human innervated engineered skin construct was made (170). After 18 days of co-culture with the skin construct, iPSC-derived neurons formed a network of neurites reaching up to the epidermis, but strikingly only when combined with Schwann cells. These neurons released neuropeptides upon stimulation, demonstrating some level of functionality. Another recent study differentiated itch sensory neuron-like cells (ISNLCs) from iPSCs and reported that these cells displayed action potentials in response to itch-specific stimuli (171). ISNLCs expressed receptors for cytokines IL-4/IL-13, which contributed to their activation. They subsequently integrated the ISNLCs into TES as a proof of principle.

As an alternative to the iPSC-derived neurons, another group utilized human-induced neural stem cells (hiNSCs) directly reprogrammed from dermal fibroblasts to innervate their skin constructs (172). The model includes a hypodermis containing patient-derived lipoaspirates and immune cells. The epidermal and dermal components are made with a novel collagen-silk gel that is then placed on top of the hypodermis. The primary focus of this study was to generate a TES that could recapitulate the neuro-immuno-cutaneous system. They found patient-specific variations in the release of cytokines in the presence of patient-derived adipose tissue, highlighting the importance of patient-specific modeling. The study did not include morphological validation of innervation and neuron organization. Although hiNSCs expressed several sensory neuron markers, the sensory neuron-specific identity and function of these cells are yet to be determined.

Co-culturing neurons in TES has also been shown to be influential on epidermal cells. Epidermal thickness and density have been reported to be higher, and the apoptosis of keratinocytes to be lower in innervated TES (160). Neurons induced keratinocyte proliferation and increased epidermal thickness via a neuropeptide, calcitonin gene-related peptide (CGRP) (162). Likewise, keratinocytes and fibroblasts were shown to regulate skin innervation via neurotrophic factors, NGF (170). These complex reciprocal interactions highlight the importance of incorporating innervation in TES to study skin physiology and pathology. Notably, such neuropeptides and neurotrophic factors, including CGRP and NGF, are closely correlated with CIPN pathology, yet how they contribute to neuron-skin crosstalk is largely unknown. These innervated models provide platforms to study how neuron-skin interactions could drive CIPN pathology. Emulating key aspects of a neuron's native environment, these models have the potential to advance *in vitro* drug screening models for future CIPN studies.

### Limitations and Future Potentials of TES for CIPN Studies

All TES models reported so far use similar innervation approaches where the vertical neurite growth and branching in the dermis were stimulated by adding growth factors into the culture medium or inclusion of other cells, such as Schwann cells. This process, unfortunately, results in a spontaneous and uncontrolled innervation. To achieve a physiologically relevant and truly functional innervated TES model, it is imperative to control the level and type of innervation. Given layer-specific targeting of sensory neurons of different modalities in the epidermis and dermis, it is also important to guide these nerves to their final end-organ, e.g., different epidermal layers vs. papillary dermis. Moreover, most of the studies discussed above do not know which subtypes of sensory neurons innervate the skin, and their function still requires validation as the innervation does not necessarily lead to sensation. In the future, with advanced biofabrication techniques such as 3D-bioprinting and novel and tunable biomaterials, it may be possible to spatially control and guide the innervation process in TES to achieve function. There is also a need to produce robust differentiation protocols to derive and characterize each specific subpopulation of sensory neurons from iPSCs to achieve a physiologically relevant model of skin sensation.

Although the iPSC-derived skin organoids approach addresses the issue of cellular diversity in TES, several issues remain, including spontaneous differentiation and cyst-like organization of cells, leading to partial anatomical relevance, e.g., inside-out morphology where the epidermis is inaccessible as it is located in the interior of the organoid. In addition, the dermis of these organoids is neural-crest derived and thus mimics the craniofacial skin, as opposed to the other sites of the human dermis, which are mesoderm-derived. With the emerging engineering approaches in the organoid field, such as cell and ECM micropatterning, some of these limitations may soon be addressed. Future studies should include validation of the level of maturation of the sensory neurons in these embryonic models to serve as a model for CIPN.

## Conclusion

To advance our understanding of CIPN pathology that could lead to effective prevention and treatment options in the clinic, future studies should consider characterizing neuronal changes in the context of their local environment. Given that surrounding non-neuronal cells are dynamically maintained and actively crosstalk with sensory neurons, experimental platforms that recapitulate neurons' local environment and could monitor real-time changes would be ideal. Currently, no single experimental model could fulfill such conditions. Yet, a combination of several models could provide significant insights and robust validation of pathological mechanisms that often fall short by using a single model approach. Together with existing rodent models of CIPN, simple *in vivo* animal models and simplified human 3D models reviewed here could provide complementary advantages that allow characterization of inter-cellular and cell-type-specific mechanisms of CIPN pathology with clear functional readouts. These models also have the potential to simulate the cancer microenvironment that would further validate the efficacy and safety of potential targets for CIPN prevention and treatment.

## Author Contributions

GS conceived the manuscript, drafted the first outline of the paper, wrote sections Introduction, Experimental Approaches to Study CIPN Mechanisms, *Drosophila* Models of CIPN (Sensory Neurons and Their Extracellular Environment in *Drosophila, Drosophila* as a Model to Study Peripheral Neuropathy, and CIPN Study Design and Readouts in *Drosophila* Models), and Conclusion, and edited and revised all sections in the manuscript. HA wrote section Possibilities of Utilizing Human-Engineered Skin Models in Understanding CIPN. MS wrote the draft of section *Drosophila* Models of CIPN (Potential CIPN Mechanisms Identified in *Drosophila* Models) and assisted with the PubMed search. All authors contributed to the article and approved the submitted version.

## Funding

GS was supported by Thompson Family Foundation Initiative for Chemotherapy-Induced Peripheral Neuropathy and Sensory Neuroscience at Columbia University.

## Conflict of Interest

The authors declare that the research was conducted in the absence of any commercial or financial relationships that could be construed as a potential conflict of interest.

## Publisher's Note

All claims expressed in this article are solely those of the authors and do not necessarily represent those of their affiliated organizations, or those of the publisher, the editors and the reviewers. Any product that may be evaluated in this article, or claim that may be made by its manufacturer, is not guaranteed or endorsed by the publisher.
